# Alterations in erythrocyte membrane transporter expression levels in type 2 diabetic patients

**DOI:** 10.1038/s41598-021-82417-8

**Published:** 2021-02-02

**Authors:** Edit Szabó, Anna Kulin, László Korányi, Botond Literáti-Nagy, Judit Cserepes, Anikó Somogyi, Balázs Sarkadi, György Várady

**Affiliations:** 1 Institute of Enzymology, ELKH Research Centre for Natural Sciences, Center of Excellence By Hungarian Academy of Sciences, Magyar tudósok körútja 2, 1117 Budapest, Hungary; 2 Drug Research Center, Balatonfüred, Hungary; 3 CellPharma Kft, Budapest, Hungary; 4 2nd Department of Internal Medicine, Semmelweis University, Budapest, Hungary; 5 Department of Biophysics and Radiation Biology, Semmelweis University, Budapest, Hungary

**Keywords:** Molecular biology, Biomarkers

## Abstract

Type 2 diabetes mellitus (T2DM) is one of the most common multifactorial diseases and several membrane transporters are involved in its development, complications and treatment. We have recently developed a flow-cytometry assay panel for the quantitative determination of red cell membrane protein levels with potential relevance in diseases. Here we report a detailed phenotypic analysis of a medium scale, clinically based study on the expression of T2DM-related membrane proteins, the GLUT1, GLUT3, MCT1, URAT1, ABCA1, ABCG2 and the PMCA4 transporters in erythrocytes. By comparing age-matched control subjects and three groups of T2DM patients (recently diagnosed, successfully managed, and patients with disease-related complications), we found significant differences in the membrane expression levels of the transporters in these groups. This is a first detailed analysis of T2DM related alterations in erythrocyte membrane transporter protein levels, and the results suggest significant changes in some of the transporter expression levels in various patient groups. By performing a further, more detailed analysis of the clinical and molecular biology parameters, these data may serve as a basis of establishing new, personalized diagnostic markers helping the prevention and treatment of type 2 diabetes.

## Introduction

Type 2 diabetes mellitus (T2DM) is a chronic metabolic disease that affects more than 400 million people worldwide^1^. Due to the insufficient insulin production and/or the tissue insulin resistance, the compensating high blood sugar levels may cause damages to the heart, blood vessels, eyes, kidneys and the nervous system^2^. Since the symptoms of T2DM in the early stages are mostly non-specific, currently there are no established biomarkers that are usable for the presymptomatic or early detection of the disease. While there are several biomarkers, including HbA1c, for the characterization of the already developed disease, the prediction of disease-related complications is still difficult. Numerous studies focus on the complex genetic background of T2DM but, according to the genome-wide association study (GWAS) results, the polymorphic variants in the human genome are difficult to interpret with a predictive value. Moreover, environmental and nutritional factors greatly influence the actual phenotypic effects of the genetic polymorphisms.

In this project we initiated a new approach to provide potential biomarkers for tracking disease progression and predicting possible complications in T2DM, by studying the diabetes-related transporters in the membranes of the red blood cells (RBC). In our previous work^3–7^ we have described a simple, rapid and reliable flow cytometry-based diagnostic assay for the quantitative analysis of the RBC membrane proteins, to examine individual variations or to compare patient groups.

The red cells are easily obtained in relatively large quantities even from a fingertip blood sample and may provide a tool to reflect general changes in membrane protein expression levels. Earlier we found a direct correlation between ABCG2 genotypes and the respective expression levels of this protein in the RBC membrane, and identified a new mutation (M71V) in *ABCG2* that significantly reduces the protein levels in the RBCs^6^. We have revealed the effects of genetic polymorphisms and mutations in the *ABCB6* gene, causing altered ABCB6 protein expression levels^4^, and found reduced calcium pump (PMCA4b) expression in the red cell membrane, caused by a minor haplotype^7^. When studying RBC membrane proteins in the multifactorial Alzheimer’s disease (AD), we found that the levels of GLUT1, INSR, ABCA1 and ABCG2 proteins were significantly reduced in late AD patients, as compared to the levels in healthy, age-matched controls. Moreover, GLUT1 and INSR expression levels were significantly decreased in the early onset AD patients^5^.

In the present study, we have investigated the alterations of several red blood cell membrane transporter proteins in T2DM. The changes in the expression levels were quantitatively analyzed in the untreated (newly diagnosed) and in the successfully managed patients, as well as in patient groups showing treatment resistance and/or disease-related complications. The investigated transporters were selected by bioinformatics and network analysis of GWAS, and included the glucose exchangers GLUT1 and GLUT3, the monocarboxylate transporter MCT1, the uric acid transporter URAT1, the cholesterol efflux regulatory protein ABCA1, the multidrug transporter ABCG2, and the active Ca^2+^ transporter PMCA4b^8–11^.

In the course of this project we have collected detailed clinical and laboratory data, as well as cellular DNA from all of the individuals entering this study. The goal of this medium-scale study was to establish potential new biomarkers for T2DM diagnostics. This paper is the first, phenotypic report on the data obtained for RBC protein expression levels and using a basic clinical classification of the patients. As shown below, in several cases we observed significant differences in the expression levels of these proteins in T2DM patients, as compared to age-matched healthy control subjects. In cases of several transporters, significant differences in RBC transporter expression were also observed among the three patient groups. Correlations of transporter alterations were found especially strong with HbA1c levels. The statistical analysis presented in this paper should serve as a basis for a further detailed examination of the molecular genetic background of the observed alterations, and for correlating individual membrane transporter expression levels with clinical and clinical laboratory data.

## Results

### General laboratory data

In the T2DM patients, the key disease-specific data showed significant differences in weight, BMI, waist circumference and HbA1c levels, compared to the control subjects. We have formed three major groups of the T2DM patients, the recently diagnosed, untreated patients, successfully managed (stable) patients in disease-free condition, and patients with disease-related complications, including patients refractory for the applied treatment. Significant differences were found in the weight, waist circumference, BMI and HbA1c values in these groups (Table 1). There were also significant differences in the blood glucose levels, blood insulin levels, HOMA tests, RBC, WBC, monocyte and eosinophil granulocyte levels between the control subjects, the untreated and the successfully managed T2DM patient groups, respectively. There were significant differences in creatine, eGFR (MDRD) and eGFR (CDK-EPI) values between successfully managed patients and those with disease-related complications (see data in Supplementary Table 2).

**Table 1:** Summary of some key data for age-matched control subjects and the three groups of T2DM patients.

	Individuals in the group (n)	Age (years)	Height (cm)	Weight (kg)	Waist circumference (cm)	BMI (kg/m^2^)	HbA1c (%)
Control	59	61 ± 12	169 ± 11	77 ± 17	95 ± 13	27 ± 5	5.3 ± 0.5
Untreated	36	64 ± 8	166 ± 12	80 ± 18	99 ± 12	29 ± 5	5.9 ± 0.3
p con/untr		0.1723	0.4071	0.3454	0.1989	0.0523	< 0.0001****
Treated	61	63 ± 10	169 ± 10	89 ± 18	109 ± 14	31 ± 5	7 ± 1.2
p con/treat		0.3674	0.7583	0.0002***	< 0.0001****	< 0.0001****	< 0.0001****
p untr/treat		0.5885	0.2765	0.0215*	0.0004***	0.0756	< 0.0001****
Complication	23	66 ± 9	168 ± 10	87 ± 15	113 ± 10	31 ± 4	8.1 ± 1.3
p con/compl		0.0392*	0.7135	0.0128*	< 0.0001****	0.0012**	< 0.0001****
p untr/compl		0.3407	0.6851	0.1352	< 0.0001****	0.1773	< 0.0001****
p treat/compl		0.1554	0.5343	0.581	0.1644	0.8144	0.0022**

The values are expressed as means ± SD. The p values were calculated by the Welch’s t-test.

### Red blood cell transporter protein levels

We have measured the expression levels of several transporters in the RBC membranes of normal healthy individuals and T2DM patients. The membrane transporters examined here were GLUT1 (SLC2A1), GLUT3 (SLC2A3), MCT1 (SLC16A1), URAT1 (SLC22A12), ABCA1, ABCG2, and PMCA4b. In all cases previous titrations assured the quantitative determination of these protein levels in the hemoglobin-free fixed ghosts of red blood cells (see^3,5^). Because of the heterogeneity of the groups examined and the potentially variable causes of membrane transporter expression, we have performed several statistical tests to evaluate the data. In addition to the distribution patterns, the significance of the differences between the groups was estimated by the Mann–Whitney *U*-test and the Kruskal–Wallis test, and the Kernel density estimation diagrams were used to visualize data distributions.

### RBC transporter levels in control subjects and T2DM patients

**Figure 1:**
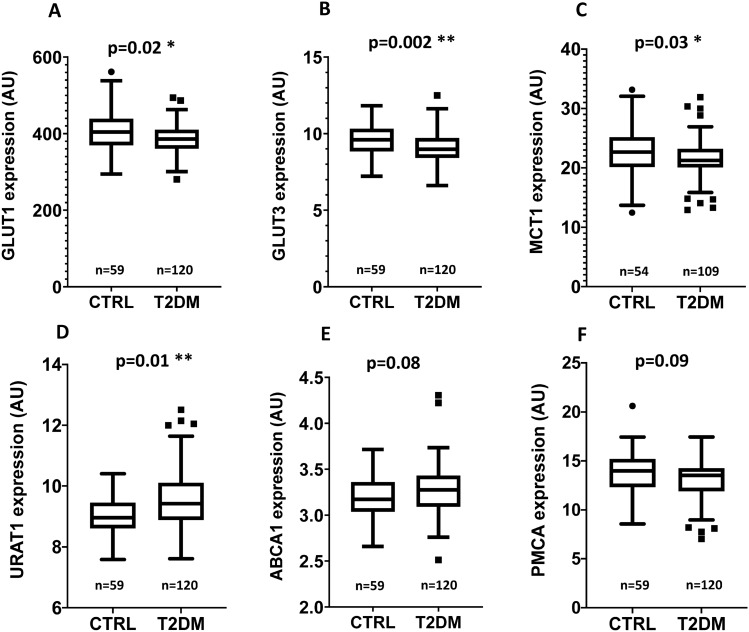
Expression of the RBC membrane proteins in age-matched control subjects and in T2DM patients, measured by flow cytometry. (**A**) GLUT1, (**B**) GLUT3, (**C**) MCT1, (**D**) URAT1, (**E**) ABCA1, (**F**) PMCA4b. The AU (arbitrary unit) values were calculated as described in the “Methods”. The p values were calculated by the Mann–Whitney test. The number of participants (n) is listed in each panel.

When examining the differences in the expression levels between control subjects and the entire T2DM patient group, we found significant differences in the expression levels of GLUT1, GLUT3, URAT1, and MCT1 (Table 2; Fig. 1). Both GLUT1, GLUT3, and MCT1 expression levels were significantly lower in the T2DM patients, while URAT1 expression was significantly increased, compared to the control subjects. In addition, although not reaching significance, a tendency of increased expression of the ABCA1 lipid transporter and a decreased expression of the plasma membrane calcium pump (PMCA4b) was observed in the group of the T2DM patients, compared to age-matched controls. No measurable alterations were observed in the expression levels of the ABCG2 transporter, and the Band-3 protein (SLC4A1) between these groups, and the level of this latter protein could serve as a stable parameter for red cell membrane preparations.

**Table 2:** Expression of RBC membrane proteins in age-matched controls and in T2DM patients, measured by flow cytometry.

	GLUT1	GLUT3	MCT1	URAT1	ABCA1	PMCA4b	ABCG2	Band3
Control	404.49 ± 55.51	9.62 ± 1.08	22.84 ± 4.32	9.19 ± 0.73	3.21 ± 0.25	13.74 ± 2.45	6.60 ± 1.29	1159.47 ± 70.17
T2DM	385.47 ± 43.60	9.09 ± 1.06	21.53 ± 3.28	9.56 ± 1.00	3.27 ± 0.27	13.18 ± 2.05	6.41 ± 1.13	1159.71 ± 60.68
M-W p value	0.0165*	0.0018**	0.026*	0.0088**	0.0814	0.0879	0.3199	0.721

The protein expression values, calculated as described in the “Methods” section, are expressed as means ± SD. The p values were calculated by the Mann–Whitney test. n = 59 for all controls, except for MCT1 (n = 54), and n = 120 for all T2DM patients, except for MCT1 (n = 109).

**Figure 2:**
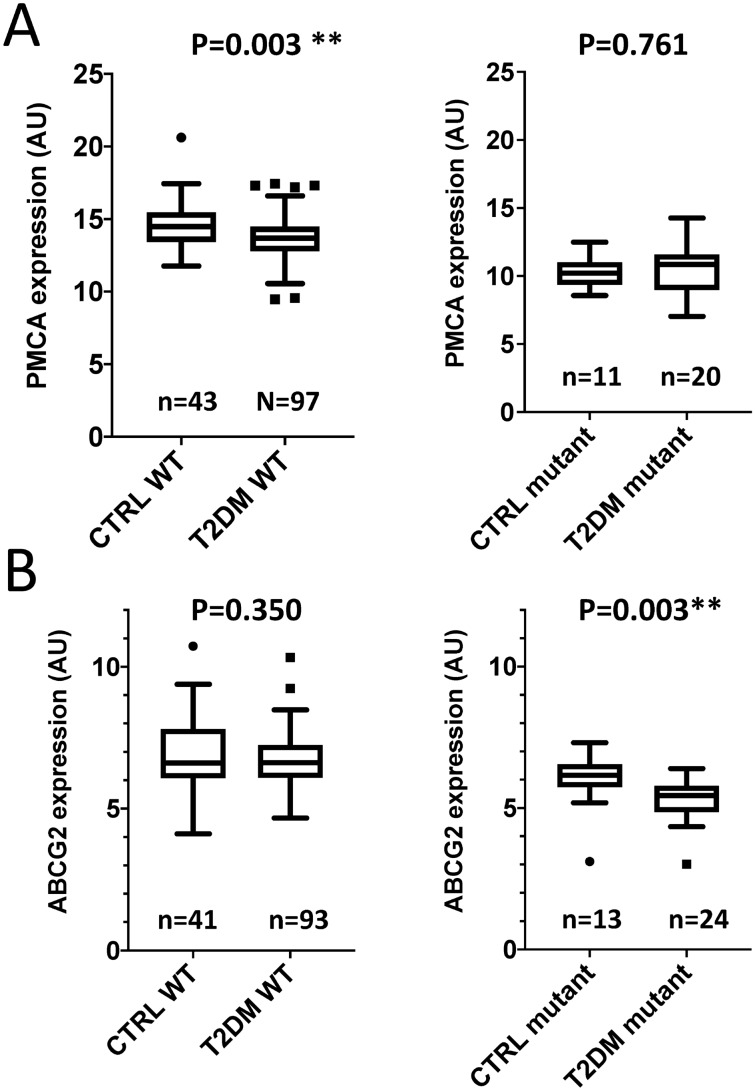
Expression of RBC membrane proteins in age-matched control subjects and in T2DM patients, having different genetic variations. The p values were analyzed by the Mann–Whitney test. (**A**) Expression of the PMCA4b protein, carrying the wild-type haplotype or the minor haplotype in the ATP2B4 gene. (**B**) Expression of the ABCG2 transporter in individuals with the wild-type ABCG2 and those with the ABCG2-Q141K variant. The number of participants (n) is indicated in each panel.

As shown in Table 2, we did not find significant differences in the PMCA4b expression between control individuals and T2DM patients. However, we have previously shown that the presence of a minor haplotype in the ATP2B4 gene (coding for PMCA4b) significantly reduces the expression level of this protein in the red cell membrane^7^. Therefore, we also performed a separate analysis and comparison of the control individuals and T2DM patients, carrying either the wild-type or the minor variant of this haplotype. As shown in Fig. 2A, in the case of individuals carrying the wild-type haplotype, the expression level of the PMCA4b protein was significantly lower in the T2DM patients compared to the control individuals. In contrast, there was no such difference in the case of individuals carrying the minor variant of the haplotype (Fig. 2A, right panel).

When measuring the RBC expression of the ABCG2 protein we did not observe a difference between control individuals and T2DM patients (see Table 2). It has been shown earlier, that the common polymorphism Q141K in *ABCG2* (rs2231142), causes lower RBC protein expression^3,6^, thus we also examined the potential effect of this polymorphism in T2DM patients. As shown in Fig. 2B, in individuals carrying the wild type *ABCG2* gene, there was no difference in the level of ABCG2 protein between control subjects and T2DM patients. However, in individuals carrying the Q141K variant, T2DM patients had significantly lower levels of RBC ABCG2 than the control subjects.

### RBC transporter levels in control subjects and in three T2DM patient groups

In the following analysis we compared the red cell membrane transporter expression levels among the control subjects, the untreated, the successfully managed T2DM patients, and the T2DM patients with disease-related complications (Table 3).

**Table 3:** Results of the Mann–Whitney analysis of membrane protein expression in control individuals and in T2DM patients grouped into recently diagnosed, untreated patients, successfully managed patients, and patients showing treatment resistance or disease-related complications.

	GLUT1	GLUT3	MCT1	URAT1	ABCA1	PMCA4b	ABCG2	Band3
Control	404.49 ± 55.51	9.62 ± 1.08	22.84 ± 4.32	9.19 ± 0.73	3.21 ± 0.25	13.74 ± 2.45	6.60 ± 1.29	1159.47 ± 70.17
Untreated	397.50 ± 51.69	9.13 ± 1.08	21.65 ± 4.06	9.07 ± 0.53	3.12 ± 0.24	13.25 ± 1.98	6.65 ± 1.28	1150.55 ± 72.82
p con/untr	0.4381	0.0329*	0.1599	0.9437	0.1121	0.2116	0.7908	0.7864
Treated	386.22 ± 37.80	9.24 ± 1.10	21.43 ± 3.19	9.62 ± 1.12	3.31 ± 0.25	13.18 ± 2.28	6.36 ± 1.12	1163.09 ± 53.27
p con/treat	0.0434*	0.0431*	0.0339*	0.0259*	0.0233*	0.2015	0.2422	0.7776
p untr/treat	0.4017	0.8599	0.9376	0.0154*	0.0003***	0.7339	0.2283	> 0.9999
Compl	364.64 ± 37.99	8.65 ± 0.81	21.59 ± 2.32	10.17 ± 0.82	3.43 ± 0.24	13.07 ± 1.53	6.19 ± 0.88	1165.08 ± 59.52
p con/compl	0.0021**	0.0002***	0.1611	< 0.0001****	0.0003*	0.1253	0.1606	0.7899
p untr/compl	0.0142*	0.0744	0.7450	< 0.0001****	< 0.0001****	0.9908	0.0942	0.9939
p treat/compl	0.0171*	0.0297*	0.6853	0.0175*	0.0679	0.4836	0.7032	0.9841

The protein expression values are expressed as means ± SD. n = 59 for all controls, except for MCT1 (n = 54), n = 36 for all recently diagnosed untreated T2DM patients, except for MCT1 (n = 31), n = 61 for all successfully managed T2DM patients except for MCT1 (n = 55), and n = 23 for all T2DM patients showing treatment resistance or disease-related complications.

Interestingly, we did not observe significant alterations in the transporter expression levels between the age-matched controls and the recently diagnosed, untreated T2DM patients, except for a decrease in the GLUT3 levels in the latter group. This could be partially explained by the relatively low number of the recently diagnosed patients in this study. In contrast, significant differences were found in the GLUT1, GLUT3, MCT1, (all decreased) the URAT1, and the ABCA1 (both increased) levels, when the successfully managed patients were compared to the control subjects. This difference was also significant for the increase of the URAT1 and the ABCA1 in the successfully managed patients compared to the untreated patients.

When analyzing the data for the T2DM patients showing disease related complications, in these cases we found a major decrease in the GLUT1 transporter level, a decrease in the GLUT3 level (controls versus T2DM complications and successfully managed patients versus those with T2DM complications), an increase in URAT1 level and in the ABCA1 level (controls versus T2DM complications, and untreated patients versus those with T2DM complications).

**Figure 3:**
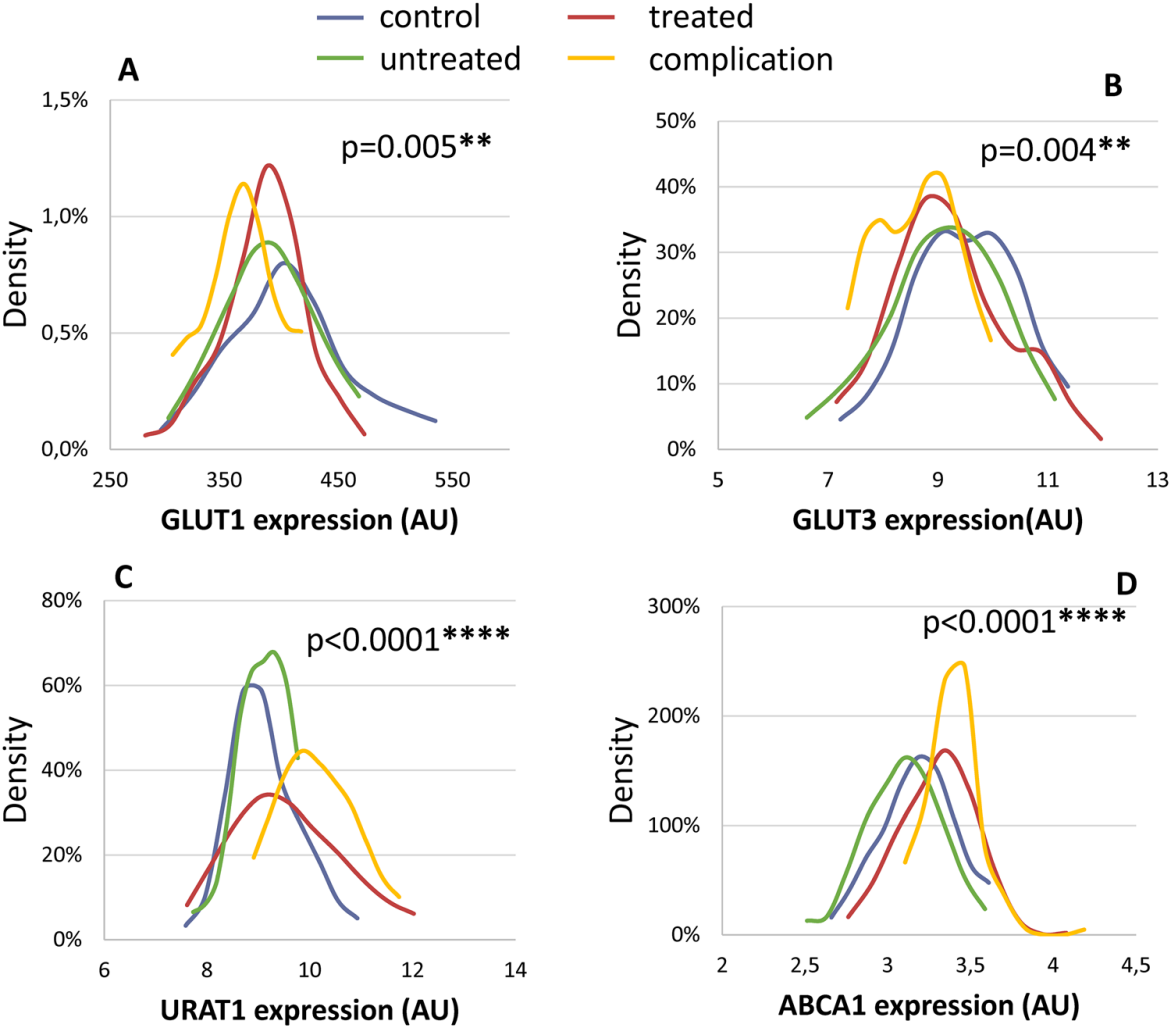
Kernel density diagrams and the p values obtained for the statistical differences between the protein expression values within the groups. The p values were analyzed by the Kruskal–Wallis test. (**A**) GLUT1; (**B**) GLUT3; (**C**) URAT1; (**D**) ABCA1. Blue: control individuals (n = 59, except in MCT1, n = 54) grey: recently diagnosed, untreated T2DM patients (n = 36, except for MCT1, n = 31), red: successfully managed patients (n = 61, except for MCT1, n = 55), and orange: patients showing treatment resistance or disease-related complications (n = 23).

In order to further analyze and visualize these differences between the T2DM patient groups and the control subjects we prepared Kernel density estimation diagrams. The statistical differences between the protein expression values of the groups were analyzed by the Kruskal–Wallis test, allowing the comparison of more than two samples. These analyses are shown in Fig. 3, for the transporter expressions in which we observed significant differences. As this representation indicates, especially the T2DM patients with disease-related complications showed a significantly different distribution curve in the evaluated transporter expression levels.

### Potential correlation between RBC transporter levels, blood glucose and HbA1c in T2DM patients

Since higher blood glucose levels are connected to T2DM pathology, in the following we have analyzed the potential relationship between actual blood glucose levels and the expression of RBC membrane transporters. We have formed three groups based on blood sugar levels according to the American Diabetes Association (ADA) recommendations in which blood sugar levels below 5.5 mmol/L are considered normal, increased levels (IL) between 5.6 and 6.9 mmol/L are considered to indicate prediabetes, while highly increased levels (HIL) at or above 7 mmol/L indicate the presence of diabetes^12^. The results of this analysis are shown in Table 4.

**Table 4:** Results of Mann–Whitney analysis of membrane protein expression values in the combined groups of controls and T2DM patients, divided into normal (n = 72, except for MCT1, n = 61), increased level (IL—n = 30, except for MCT1, n = 28) and highly increased level (HIL—n = 18, except for MCT1, n = 15) groups, based on blood glucose levels.

Blood glucose	GLUT1	GLUT3	MCT1	URAT1	ABCA1	PMCA4b	ABCG2	Band3
Normal	407.93 ± 54.33	9.47 ± 1.13	22.32 ± 4.29	9.08 ± 0.62	3.16 ± 0.26	13.65 ± 2.24	6.67 ± 1.30	1155.33 ± 73.31
IL	395.06 ± 38.68	9.32 ± 1.07	22.01 ± 4.77	8.91 ± 0.50	3.20 ± 0.20	13.45 ± 1.80	6.39 ± 1.04	1153.99 ± 59.67
HIL	392.19 ± 37.14	9.59 ± 1.16	21.82 ± 4.33	9.02 ± 0.49	3.23 ± 0.25	12.21 ± 2.56	6.43 ± 1.04	1164.31 ± 60.29

p values	GLUT1	GLUT3	MCT1	URAT1	ABCA1	PMCA4b	ABCG2	Band3
Norm/IL	0.4276	0.4481	0.8658	0.4491	0.4116	0.3065	0.5142	0.8294
Norm/HIL	0.3283	0.6186	0.5961	0.7699	0.3026	0.0477*	0.4214	0.9005
IL/HIL	0.7922	0.3834	0.7960	0.3495	0.6768	0.4314	0.8125	0.9412

The protein expression values are expressed as means ± SD.

As documented in this table, we found no correlation between the actual blood glucose levels and the RBC expression levels of any of the transporters examined. However, (unexpectedly) a decreased PMCA level was observed when comparing the subjects with normal and diabetic levels of blood glucose, respectively.

Since HbA1c values reflect a long-term presence of higher blood sugar levels, in the following analysis the T2DM patients were grouped by HbA1c levels. According to the ADA recommendations, HbA1c levels below 5.6% are regarded normal, increased levels (IL) between 5.7 and 6.4% indicate prediabetes, and highly increased levels (HIL) above 6.5% are characteristic for diabetic patients^12^. As shown in Table 5, higher HbA1c levels correlated with significantly reduced GLUT1, GLUT3, and MCT1 expression levels in the red cell membrane, while URAT1, and ABCA1 expression levels were increased in patients with higher HbA1c. This correlation was especially strong when transporter expression values in subjects with normal HbA1c were compared with those in patients with highly elevated HbA1c levels. In this analysis reduced ABCG2 transporter levels were also observed.

**Table 5:** Results of Mann–Whitney analysis of membrane protein expression levels in the combined groups of controls and T2DM patients, divided into normal (n = 55, except for MCT1, n = 50), increased level (IL—n = 52, except for MCT1, n = 46) and highly increased level (HIL—n = 64, except for MCT1, n = 59) groups, based on HbA1c levels.

HbA1c	GLUT1	GLUT3	MCT1	URAT1	ABCA1	PMCA4b	ABCG2	Band3
NORMAL	405.55 ± 52.72	9.63 ± 1.08	23.02 ± 4.45	9.05 ± 0.61	3.21 ± 0.26	13.59 ± 2.27	6.63 ± 1.21	1152.75 ± 70.53
IL	394.98 ± 46.63	9.17 ± 1.11	21.28 ± 4.03	9.20 ± 0.78	3.16 ± 0.25	13.36 ± 1.96	6.62 ± 1.34	1156.82 ± 64.62
HIL	378.88 ± 40.02	9.00 ± 1.05	21.64 ± 2.67	9.86 ± 1,04	3.35 ± 0.25	13.03 ± 2.19	6.22 ± 0.89	1163.07 ± 55.39

p values	GLUT1	GLUT3	MCT1	URAT1	ABCA1	PMCA4b	ABCG2	Band3
Norm/IL	0.2752	0.0197*	0.0335*	0.3117	0.3937	0.4131	0.7574	0.7398
Norm/HIL	0.0045**	0.0019**	0.0226*	< 0.0001****	0.0025**	0.1533	0.0251*	0.9133
IL/HIL	0.0426*	0.3826	0.7103	0.0006***	< 0.0001****	0.7223	0.0897	0.6284

The protein expression values are expressed as means ± SD.

**Figure 4:**
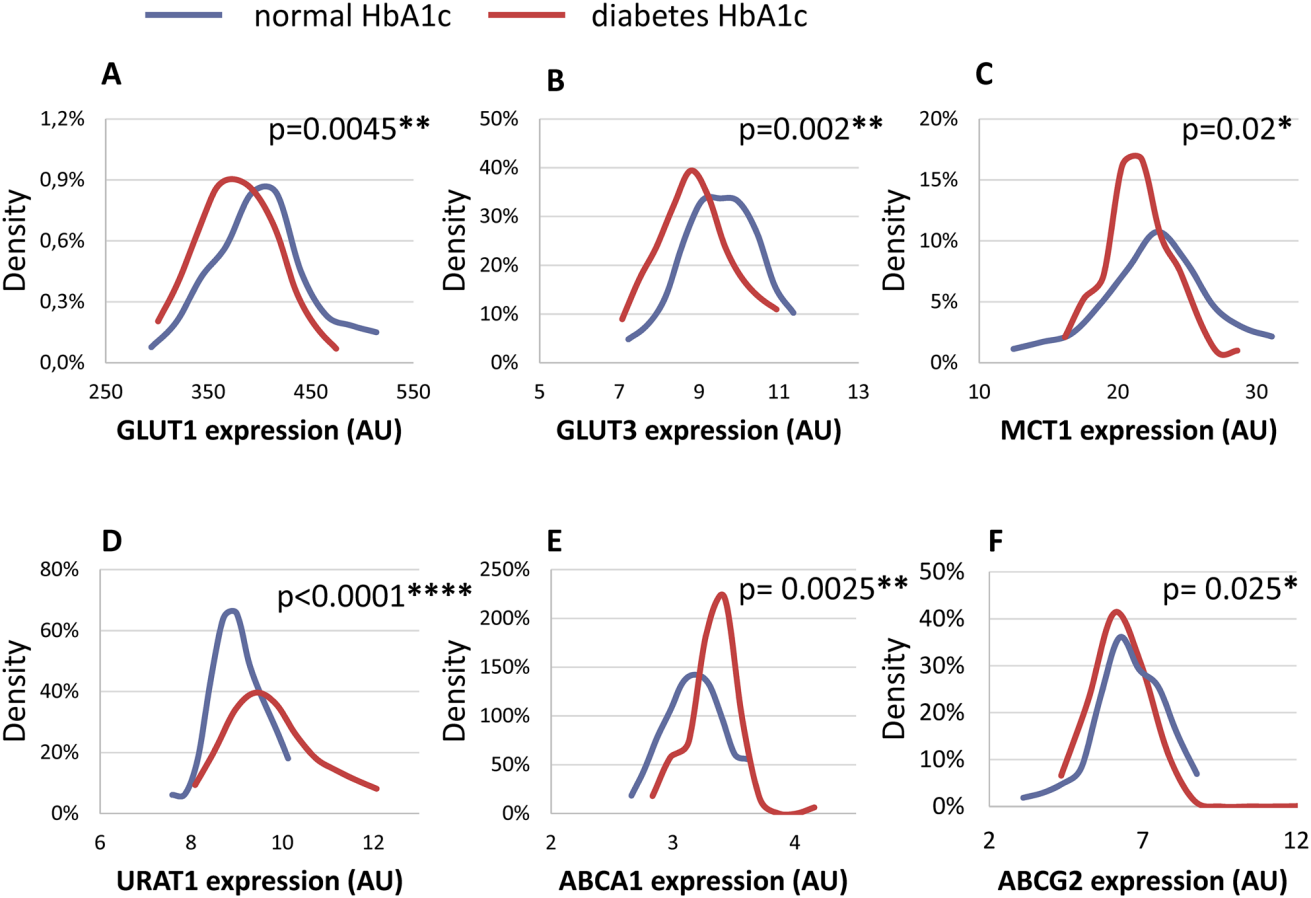
Kernel density diagrams and the p values obtained for the statistical differences between the protein expression levels within the groups with normal HbA1c (n = 55, except for MCT1, n = 50), versus highly increased HbA1c levels (HIL—n = 64, except for MCT1, n = 59). Blue: normal, < 5.6% HbA1c, red: HIL, > 6.5% HbA1c. The p values were calculated by Mann–Whitney test. Expression levels of (**A**) GLUT1; (**B**) GLUT3; (**C**) MCT1; (**D**) URAT1; (**E**) ABCA1, and (**F**) ABCG2.

The Fig. 4 shows the Kernel density diagrams and the p values obtained for the statistical differences between the protein expression values within the groups with normal HbA1c versus highly increased levels (HIL) of HbA1c. As shown in this Figure, the density distributions clearly indicate major differences in the distribution of the RBC expression levels between the subjects with normal, or highly elevated HbA1c.

## Discussion

Membrane proteins play a key role in numerous phases of the development of T2DM. Studies related to the expression and function of the metabolite or ion transporters, as well as the membrane proteins affecting drug treatment of the disease, may provide new diagnostic tools in promoting the prevention and/or the efficient treatment of T2DM. Quantitative flow cytometry measurement of RBC membrane proteins is a relatively quick and cost-effective method, which requires only a small amount (50–200 µL) of blood. The method we have developed provides reliable data on RBC protein levels, which can be used to examine the potential correlations with clinical conditions and eventually find the genetic background of altered protein expression levels.

In the present paper we performed a basic, phenotypic analysis for finding potential correlations between the RBC expression levels of several disease-relevant membrane transporters in T2DM patients and compared these to the levels found in matched control subjects. Here we have studied the RBC expression levels of the GLUT1, GLUT3, MCT1, URAT1, ABCA1, ABCG2, and the PMCA4 proteins.

GLUT1 (SLC2A1) is the main glucose transporter of the red blood cell and is present in large quantities in many other tissues (e.g., blood–brain barrier, adipocytes, kidney cortex mesangial cells). The regulation of GLUT1 expression is influenced by both external and internal factors, e.g., blood glucose levels, hormones, or hypoxia modulate the expression level of this protein in various organs or tissues^13–16^. GLUT3 (SLC2A3) is the main glucose transporter in the central nervous system and this protein is also present in other tissues. There are only limited data on the regulation of GLUT3 expression in T2DM, while it has been suggested that epigenetic alterations may result in different GLUT3 expression levels^17,18^.

The major monocarboxylate transporter in the RBC membrane is MCT1 (SLC16A1), and members of the SLC16A family are proton-dependent monocarboxylate transporters, pumping protons and monocarboxylates (*e.g.,* lactate or pyruvate) as symporters from the extracellular matrix into the cells. These proteins play an important role in the Cory cycle, that is in the metabolic pathway in which lactate produced by glycolysis in the muscle or other cells, including the red blood cells, and then lactate is converted back to glucose in the liver^9,19^. Plasma lactate concentration correlates with the rate of glycolysis reflecting mitochondrial oxidative capacity, and lower oxidative capacity is strongly associated with T2DM^20^.

URAT1 (SLC22A12) is a member of the OAT (Organic Anion Transporter) family, playing a key role in uric acid homeostasis, and present in relatively large quantities in the RBC membrane. The human-specific relatively high levels of circulating uric acid on one hand prevent bone mineral loss and oxidative damage, while on the other hand, excess uric acid leads to gout, promotes hypertension and cardiovascular disease^10^. Reduction of URAT1 levels have been reported in diabetic mice models, probably connected to insulin depletion^21,22^.

ABCA1 is a large, heavily glycosylated member of the ATP-binding Cassette (ABC) membrane transporter family, with a major role in cholesterol and phospholipid regulation in numerous tissues including the liver. ABCA1 is involved in the formation of HDL particles by promoting cholesterol transfer from the cells to apolipoprotein A. Reduction in ABCA1 expression, especially in macrophages, may promote atherosclerosis and cholesterol deposition in peripheral tissues and organs. Since HDL levels are risk factors in diabetes, ABCA1 may serve as a diagnostic marker in T2DM^5,23,24^.

Another member of the ABC family, ABCG2 (BCRP, MXR) transports endo- and xenobiotics and is one of the key uric acid transporters, responsible for most of the extrarenal, mostly enteral extrusion of urate. In addition, several statins, used in the therapy of hypercholesterinemia, hypertension, and potentially in T2DM, are also transported substrates of ABCG2, and reduced ABCG2 levels may significantly alter statin absorption and cellular statin levels (see^25^). ABCG2 is present in significant amounts in the membranes of human red blood cells, contributing to the Jr blood group^26^.

PMCA4b has a key role in numerous tissues, especially in RBCs, in maintaining the low cellular Ca^2+^ concentrations^27,28^. Under certain pathological conditions, including *e.g.,* hereditary hemolytic anemia, malaria, and also diabetes mellitus, the Ca^2+^ levels in the RBCs have been reported to increase^29,30^, due to a decreased PMCA activity^31,32^.

In the present, basic phenotypic study we found significant disease, or disease-complications related alterations in all these transporter levels examined in the erythrocyte membranes. GLUT1, GLUT3, and MCT1 expression levels decreased in the general group of T2DM patients (Table 2), and this decrease was especially significant when the control or recently diagnosed patients were compared to successfully managed patients or to patients with disease-related complications (see Table 3, Figs. 1 and 3). Similarly, a significant increase in the RBC expression of the URAT1 transporter was observed under all these conditions, while a significantly increased ABCA1 expression was only found when successfully managed patients or patients with disease-related complications were compared to control subjects or untreated T2DM patients (Table 3).

In these general comparisons, PMCA4b, and ABCG2 levels did not show significant alterations, while, interestingly, if the subgroups of control individuals and T2DM patient carrying polymorphic variants of these proteins were compared, significant differences were found. That is, PMCA4b expression was lower in T2DM patients with the wild-type haplotype than in control individuals with the same haplotype, whereas in patients and controls carrying the minor haplotype, there was no such difference (Fig. 2A). In contrast, in the case of ABCG2, in individuals carrying the wild type gene, there was no difference in the level of RBC protein expression between control subjects and T2DM patients. However, in individuals carrying the Q141K polymorphic variant which has a processing problem, T2DM patients had significantly lower levels of RBC ABCG2 than the control subjects (Fig. 2B).

In the following we examined the correlations of the RBC membrane transporter expression levels with blood glucose and HbA1c levels, as key laboratory parameters relevant in T2DM. Interestingly, there was practically no correlation of the transporter expression levels with the actual blood glucose levels (see Table 4—except for a decrease in PMCA4b levels at high blood glucose levels), while a close correlation between decreased GLUT1, GLUT3, MCT1, and ABCG2 expression, and increased URAT1 and ABCA1 expression was observed at increasing HbA1c levels (Table 5). The Kernel density plots clearly showed these major population changes relevant to HbA1c levels (Fig. 4). These data strongly suggest that the RBC membrane protein expression levels correlate with relatively long-term changes, either evoked by genetic variations or by permanent presence of the diabetic conditions, e.g., higher blood glucose reflected by HbA1c levels. In order to explore the effect of the genetic versus metabolic conditions we plan to study families of the T2DM patients available at the register of DRC.

The membrane protein levels measured in the red cell membrane may not provide generally relevant information on protein expression in other, sometimes more relevant tissues. However, the ease of obtaining and studying RBC samples, and the increased need for blood-based biomarkers may support this approach as a valuable shortcut. The method we have developed and described in this paper regarding a T2DM study, provides reliable data on RBC protein levels, and the analysis presented here should serve as a basis for a further detailed examination of the relevant clinical and molecular biology background of the alterations in protein levels. As suggested by our previous results^3–7^, the combination of the RBC membrane protein level measurements and the relevant molecular genetic studies allow for the proper interpretation of the phenotypic changes in transport properties and functions.

In our further work we plan to establish correlations between RBC membrane protein expression levels and mutations or polymorphisms in the relevant genes. This should allow for the use of relatively simple (e.g., SNP) assays to identify disease-associated protein expression variations. In the present project the phenotypic analysis presented here, and the currently ongoing molecular genetic studies are hoped to generate a personalized diagnostic approach applying disease-associated membrane proteins. While data for individual membrane protein expression levels are not properly informative as yet in this complex disease at the clinic, the combined analysis of such biomarkers in the future should facilitate an early detection, personalized treatment, and even prediction of T2DM development.

## Methods

### Description of control and patient populations; diagnostic laboratory assays

The T2DM samples were obtained from the Drug Research Centre (DRC, Balatonfüred, Hungary) and the 2nd Department of Internal Medicine, Semmelweis University (SE, Budapest, Hungary). Both DRC and SE provided samples of control subjects, untreated and treated patients, while only SE provided samples of patients with disease-related complications. Initial examination of T2DM patients included the evaluation of personal and family medical history, as well as basic laboratory examinations.

The key laboratory diagnostic parameters for T2DM in DRC included HbA1c, glucose, and insulin levels, HOMA indices, RBC and WBC, as well as neutrophil, lymphocyte, monocyte, eosinophil, basophil, and platelet counts (Supplementary Table 2A). Laboratory data for the patients from SE also included creatine, eGFR (MDRD), eGFR (CDK-Epi) values (Supplementary Table 2B). Anthropometric data for all patients were weight, height, BMI, and waist circumference. The clinical diagnosis of T2DM was established according to the criteria of the American Diabetes Association (ADA). The major disease-related complications were neuropathy (17%), retinopathy (30%) and nephropathy (48%). Medication parameters were also recorded for the patients in the groups studied. The study was approved by the Scientific and Research Committee of the Medical Research Council, Hungary (ETT TUKEB references: 19680-3/2019/EKU, 2367-1/2019/EKU). All methods were performed in accordance with the relevant guidelines and regulations. All control subjects and patients in the study gave informed consent to participate in the research, and informed consent was obtained from all participants in this study.

### Flow cytometry

Whole blood samples were obtained from 34 untreated, 61 successfully managed (compensated) patients, and from 23 patients with disease-related complications. 61 age-matched control subjects were also included in the red blood cell protein expression study. The RBC membrane protein expression levels were measured by our recently developed method (for details see refs.^3,5^). RBC membranes were fixed by using 1% formaldehyde solution, the resulting RBC ghosts were first labeled by Alexa Fluor 647 (AF647) conjugated wheat germ agglutinin (WGA-AF647, Thermo Fisher ab3379), then incubated with the selected transporter-specific primary antibody (Supplementary Table 1). In the case of each primary antibody a titration curve was obtained to ensure maximum labeling for quantitative assay.

For fluorescence labeling, a secondary, Alexa Fluor 488-labeled (AF488) goat anti-mouse (H + L) antibody (Thermo Fisher, A11001), or in the case of SLC22A12, Alexa Fluor 488-labeled gout anti-rabbit (H + L) antibody (Thermo Fisher, A-11008) was used, in 96 well plates. RBC ghosts were analyzed for antibody staining by Attune NxT acoustic flow cytometer (excitation wavelengths: 488 nm, emission filters: 530/30 nm for AF488 and excitation wavelengths: 637 nm, emission filters: 670/14, for AF647). Secondary antibodies were also titrated to provide maximum labeling. The primary antibodies used in the RBC protein measurements are listed in Supplementary Table 1.

### SNP analysis for ATP2B4 and ABCG2

Genomic DNA was purifed from 300 µL of EDTA-anticoagulated blood samples with Puregene Blood Kit (Qiagen). TaqMan-based qPCR reactions for SNP detection were performed in a StepOnePlus device (Applied Biosystems) with premade assay mixes (ATP2B4 minor haplotype (rs1541252); cat. 4351379 and ABCG2-Q141K (rs2231142); cat. 436269) and a master mix (cat. 4371353) from Thermo Fisher. TaqMan probe specificity was verified by sequencing.

### Statistics

The differences of the key data for age-matched control subjects and the three groups of T2DM patients were analyzed by Welch’s t-test.

The AU values (Arbitrary Units for relative protein expression) were calculated by the median fluorescence values obtained with the primary + secondary antibodies together, divided by the values obtained using the secondary antibody only (for details see refs.^5^). The differences between the protein expression values of the groups were analyzed by Mann–Whitney U-test (2 samples) or Kruskal–Wallis test (more than two samples) (GraphPad Prism 8.0.1). The Mann–Whitney *U* test was used because the distribution of the data in the majority of the cases was lognormal, thus the commonly used basic t-test could not be properly applied. The dispersion of the data in the groups and the Khi-square test were calculated by Microsoft Excel 2016. The stars beside the actual p values represent significance at different levels: *p < 0.05, **p < 0.01, ***p < 0.001, ****p < 0.0001. The number of patients (n) involved in each analysis is indicated in the respective table and figure legends. The diagrams of Kernel density estimations were obtained by using NumXL (PARSON). The protein expression values are expressed as means ± standard deviation (SD).

## Supplementary Information

Supplementary Information.

## Data Availability

The datasets generated and analyzed during the current study are available from the corresponding authors on reasonable request.
