# Supplementary material for: Alterations in erythrocyte membrane transporter expression levels in type 2 diabetic patients
Source: Sci Rep. 2021 Feb 2;11:2765. doi: 10.1038/s41598-021-82417-8 (PMC7854743; doi:10.1038/s41598-021-82417-8)
Supplement: Supplementary file 1 — Supplementary Information. [file 41598_2021_82417_MOESM1_ESM.pdf]

Alterations in erythrocyte membrane transporter expression levels in type 2 diabetic patients

Edit Szabó, Anna Kulin, László Korányi, Botond Literáti-Nagy, Judit Cserepes, Anikó Somogyi, Balázs Sarkadi and György Várady

Supplementary materials

***Supplementary Table 1**. Summary of antibodies used for flow cytometry measurements of the membrane protein levels in RBCs.*

Protein	Clone	Industry	Cat. Num
PMCA4b	JA3	Merck	MABN1801
ABCG2	Bxp34	Abcam	ab3379
GLUT1	5B12.3	Merck	MABS132
GLUT3	# 202017	Bio-Techne	MAB1415
MCT1	P14612	Thermo Fisher	MA518288
ABCA1	ABH10	Merck	MAB10005
URAT1	EPR1231	Abcam	ab181237
BAND3	BIII 136	Thermo Fisher	MA120211

***Supplementary Table 2**. Summary of some key laboratory data for T2DM patients and age-matched control subjects. The values are expressed as means +/- SD. The p values were calculated by Welch's t-test.*

A: The key laboratory diagnostic parameters for T2DM in DRC included glucose, and insulin levels, HOMA indices, RBC and WBC, as well as neutrophil, lymphocyte, monocyte, eosinophil, basophil, and platelet counts. n=52 for all controls, n=36 for all recently diagnosed untreated T2DM patients, n=34 for all successfully managed T2DM patients.

B: Laboratory data for the patients from SE also included creatine, eGFR (MDRD), eGFR (CDK-Epi) values. n=29 for all successfully managed T2DM patients, and n=23 for all T2DM patients showing treatment resistance or disease-related complications.

A

Patients from DRC	glucose mmol/l	insulin mlU/l	HOMA -	RBC T/L	WBC G/L	Neutro %	Lympho %	Mono %	Eosino %	Baso %	PLT G/L
**control**	5.01+/-0.47	7.84+/-4.79	1.84+/-1.29	4.65+/-0.37	6.0+/-1.6	57.79+/-7.70	30.71+/-9.95	6.63+/-1.29	3.01+/-1.86	0.56+/-0.28	239+/-60
**untreated**	5.39+/-0.58	9.51+/-4.80	2.39+/-1.41	4.90+/-0.44	6.9+/-2.2	57.64+/-11.77	29.00+/-6.77	6.27+/-1.55	3.10+/-1.88	0.58+/-0.34	245+/-52
**p con/untr**	**0.0019****	0.115	0.0689	**0.0069****	0.0506	0.9485	0.3417	0.2476	0.8316	0.7821	0.5936
**treated**	7.66+/-2.55	10.26+/-4.81	3.57+/-2.08	4.82+/-0.32	7.3+/-2.3	58.34+/-7.50	30.95+/-6.47	5.98+/-1.32	2.32+/-1.13	0.53+/-0.25	244+/-51
**p con/treat**	**<0.0001******	**0.0281***	**0.0001*****	**0.0392***	**0.0067****	0.7458	0.8942	**0.0297***	**0.0352***	0.6043	0.6727
**p untr/treat**	**<0.0001******	0.5239	**0.0092****	0.3409	0.4108	0.7688	0.2307	0.4104	**0.0392***	0.4912	0.9206
**lab.ref.**	3.8-6	3-25	0-2	4.5-5.9	4.1-11.3	50-70	25-40	2-9	1-4	0-1	150-450

B

Patients from SE	Creatine µmol	eGFR (MDRD) ml/min/1.73m^2	eGFR (CDK-EPI) ml/min/1.73m^2
**treated**	72.0+/-16.5	88+/-22	87+/-17
**complication**	89.6+/-30.7	73+/-27	72+/-22
**p treat/compl**	**0.0186***	**0.0338***	**0.0105***
**lab.ref.**	44-99	> 60	> 60

Supplementary Figure 1 *– individual levels of GLUT1, GLUT3 and MCT1 membrane protein expression in control individuals and in T2DM patients, grouped into recently diagnosed untreated patients, successfully managed patients, and patients showing treatment resistance or disease-related complications The number of participants (n) is indicated in each panel.*

Panel A: GLUT1 expression levels
Panel B: GLUT3 expression levels
Panel C: MCT1 expression levels

A

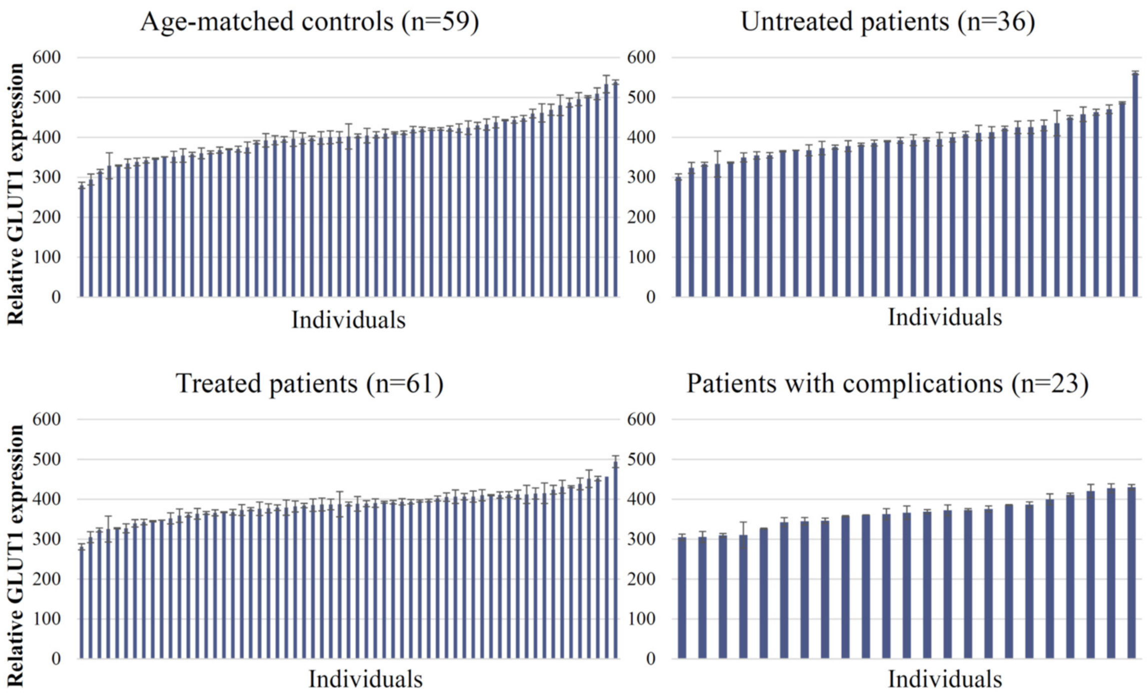

B

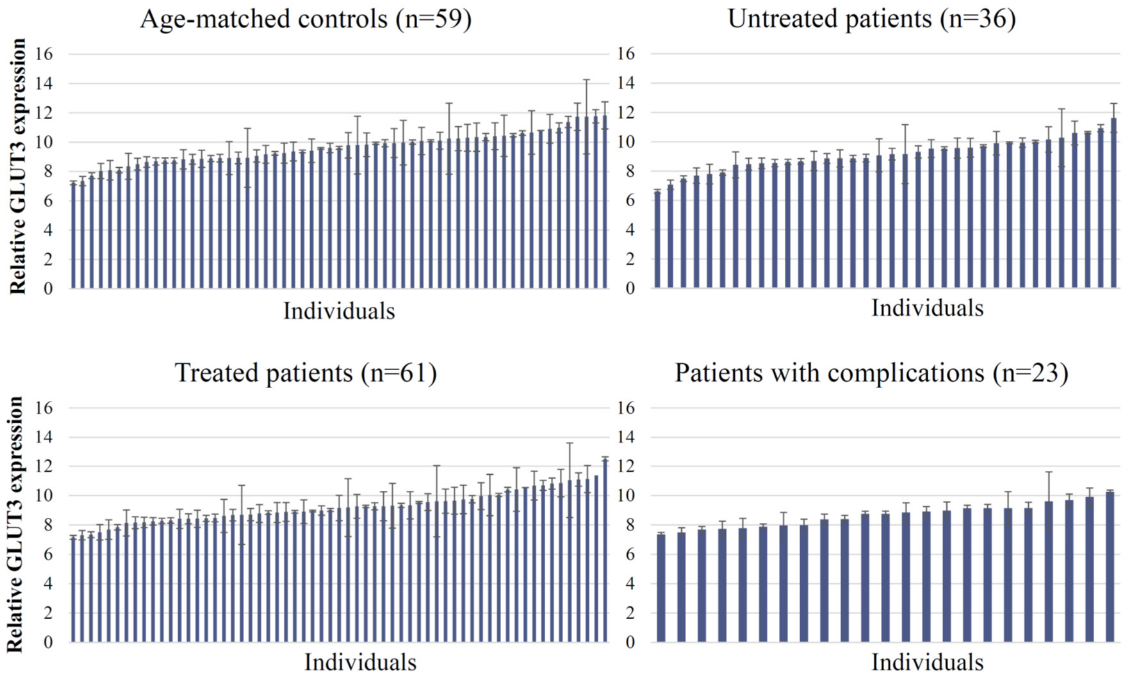
Age-matched controls (n=59)
Untreated patients (n=36)
Treated patients (n=61)
Patients with complications (n=23)
Relative GLUT3 expression
Individuals
0 2 4 6 8 10 12 14 16

C

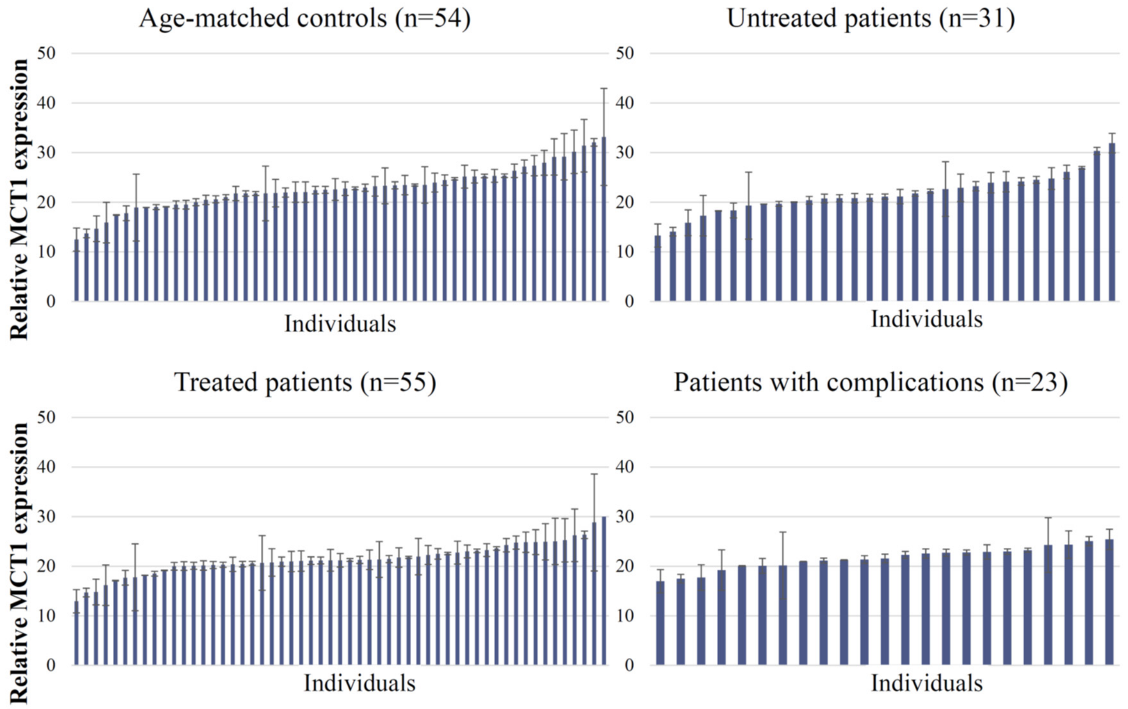
Age-matched controls (n=54)
Untreated patients (n=31)
Treated patients (n=55)
Patients with complications (n=23)
Relative MCT1 expression
Individuals
0 10 20 30 40 50
